# Inheritance and identification of molecular markers associated with a novel dwarfing gene in barley

**DOI:** 10.1186/1471-2156-11-89

**Published:** 2010-10-08

**Authors:** Xifeng Ren, Dongfa Sun, Weiwei Guan, Genlou Sun, Chengdao Li

**Affiliations:** 1 College of Plant Science and Technology, Huazhong Agricultural University, Wuhan, 430070, China; 2 Biology Department, Saint Mary's University, 923 Robie Street, Halifax, NS, B3H3C3, Canada; 3 Department of Agriculture & Food/Agricultural Research Western Australia, 3 Baron-Hay Court, South Perth, WA 6155, Australia

## Abstract

**Background:**

Dwarfing genes have widely been used in barley breeding program. More than 30 types of dwarfs or semidwarfs have been reported, but a few has been exploited in barley breeding because pleiotropic effects of dwarfing genes cause some undesired traits. The plant architecture of newly discovered dwarfing germplasm "Huaai 11" consisted of desirable agronomic traits such as shortened stature and early maturity. Genetic factor controlling the plant height in dwarf line Huaai 11 was investigated.

**Results:**

The Huaai 11 was crossed with tall varieties Monker, Mpyt, Zhenongda 3, Zaoshu 3, Advance, Huadamai 1, Huadamai 6, Hyproly and Ris01508. All the F_1 _plants displayed tall trait. Both tall and dwarf plants appeared in all the F_2 _populations with a 3:1 segregation ratio, suggesting that dwarfism of Huaai 11 is controlled by a single recessive gene, *btwd1*. Allelism test indicated that this dwarfing gene in the Huaai 11 is nonallelic with the gene *br*, *uzu*, *sdw1 *and *denso*. Using a double haploid population derived from a cross of Huadamai 6 and Huaai 11 and SSR markers the novel dwarfing gene was mapped onto the long arm of chromosome 7H, and closely linked to Bmac031 and Bmac167 with genetic distance of 2.2 cM.

**Conclusion:**

Huaai 11 is a new source of dwarf for broadening the genetic base of dwarfism. This dwarf source was controlled by a recessive dwarfing gene *btwd1*, was mapped onto the long arm of chromosome 7H.

## Background

Dwarfism is a valuable trait in crop breeding, because it increases lodging resistance and decreases damages due to wind and rain [1]. Successful use of a dwarfing gene is critical for developing dwarf cultivars [2]. In barley, more than 30 types of dwarfs or semidwarfs have been found including 'breviaristatum-*ari*', 'brachytic-*br*', 'curly dwarf-*cud*', 'denso dwarf-*denso*', 'erectoides-*ert*', 'lazy dwarf-*lzd*', 'many noded dwarf-*mnd*', 'narrow leaf dwarf-*nld*', 'semidwarf-*sdw*', 'single node dwarf-*sid*', 'slender dwarf-*sld*', '*uzu *or semibrachytic-*uzu*' and 'vegetative dwarf-*dwf*' [2-5]. However, only a few of them have successfully been used in barley breeding program. The dwarfing gene *uzu *on the chromosome 3HL has widely been used in barley breeding in China, Japan and Korea peninsula [6-10]. The dwarfing gene *sdw1 *(named as *sdw *previously) and *denso *have been used in feed barley breeding in North America and Australia [11-13], and in barley breeding in European [2,13], respectively. The *sdw1 *and *denso *genes were allelic [14-17], and were mapped onto the same region of chromosome 3HL and tagged with a RFLP marker MWG 847 by 0.6 cM [12,18,19].

Some of the dwarfing genes have rarely or never been used in barley breeding programs, although have been genetically characterized. For example, the dwarfing gene *GPert *or *ari-e *was mapped on chromosome 5H [5,20,21]. The recessive GA_3_-insensitive dwarfing genes *Rht-H1 *and *Dwf2 *were mapped on the centromeric region of chromosome 2H [22], and on the short arm of 4H [16,23], respectively. Two recessive dwarfing mutants, *gai *and *gal *were mapped on the centromere region and on the long arm of the 2H, respectively [24].

In 1993, we found a dwarf individual in field of barley landrace 'Daofu Bai Qing Ke' in Daofu County, Sichuan. Plant height of 'Daofu Bai Qing Ke' is about 119 cm, while height of the dwarf individual is only about 40 cm. The dwarf individual is naked six-rowed barley, and its maturation date is about 4 days earlier than 'Daofu Bai Qing Ke'. Multi-year and -location experiments showed that the dwarfism is stable. The dwarf line was named as 'Huaai 11'.

Genetic factor controlling plant height of the barley dwarf line Huaai 11 was investigated in the present study. Here we reported the results of genetic analysis and molecular mapping of this novel dwarfism gene.

## Methods

### Plant materials and field experiments

Plant height of Huaai 11 and other four agronomic traits were evaluated for seven years at two locations (Yaan, Sichuan Province, and Wuhan, Hubei Province). The results showed that dwarfism is constantly expressed with height of 40.8 ± 1.84 cm (Table 1). Its maturity is about 7 days earlier than the control Zaoshu 3 (early maturity variety).

**Table 1:** Evaluation of plant height and other agronomic traits of Huaai 11 in different years and locations (Yaan, Sichuan Province and Wuhan, Hubei Province)

Year	Location	Height (cm)	Days from Seedling emerging to heading	No. of Spikelets	No. of spikes per plant	Weight per1000 grains
1993-1994	Yaan	39.9	138	56	4.8	26.5
1994-1995	Wuhan	41.4	141	54	5.1	25.7
1996-1997	Wuhan	41.8	142	61	5.5	23.6
1997-1998	Wuhan	43.2	140	56	6.1	24.7
2007-2008	Wuhan	37.8	132	48	5.0	24.1
2008-2009	Wuhan	39.5	127	54	8.6	24.9
2009-2010	Wuhan	42.1	138	63	10.8	25.4
Mean		40.8	136.9	56	6.6	25.0
SD		1.84	5.43	4.933	2.28	0.98

For genetic analysis, Huaai 11 was crossed with tall varieties Monker, Mpyt, Zhenongda 3, Zaoshu 3, and Advance. The 5 F_2 _populations, their F_1 _and parents were planted in field in 1998. Additional 4 tall varieties, Huadamai 1, Huadamai 6, Hyproly and Ris01508 were crossed with Huaai 11 in year 2000. The 9 F_2 _populations, their F_1 _and parents were planted in 2002 (Table 2).

**Table 2:** Plant height in the F_2 _populations derived from the crosses between Huaai 11 and several tall cultivars

Cross	year	**No. of F**_**1 **_**plants examined**	**Plant height of F**_**1 **_**(cm)**	**No. of F**_**2 **_**plants**	**No. of F**_**2 **_**dwarf plants (< 60 cm)**	**No. of F**_**2 **_**tall plants (≥60 cm)**	**F**_**2 **_**χ**^**2 **^**(3:1)**
**Huaai 11**/Monker	1998	15	97 ± 1.13	215	52	163	0.59
**Huaai 11**/Mpyt	1998	17	95 ± 1.16	183	45	138	0.02
**Huaai 11**/Zhenongda 3	1998	13	99 ± 1.21	257	75	182	2.40
**Huaai 11**/Zaoshu 3	1998	15	102 ± 1.05	217	65	152	2.84
**Huaai 11**/Advance	1998	11	99 ± 1.09	211	57	154	0.46
**Huaai 11**/Monker	2002	13	98 ± 1.20	223	48	175	1.44
**Huaai 11**/Mpyt	2002	11	96 ± 1.09	161	45	116	0.75
**Huaai 11**/Zhenongda 3	2002	12	99 ± 1.12	259	57	202	1.24
**Huaai 11**/Zaoshu 3	2002	15	105 ± 1.23	215	50	175	0.35
**Huaai 11**/Advance	2002	15	101 ± 1.28	195	37	158	3.78
**Huaai 11**/Huadamai 1	2002	12	98 ± 1.05	242	56	186	0.45
**Huaai 11**/Huadamai 6	2002	12	87 ± 1.16	192	45	147	0.25
**Huaai 11**/Hyproly	2002	13	111 ± 1.10	196	41	155	1.74
**Huaai 11**/Ris01508	2002	15	110 ± 1.30	205	55	150	0.36

χ^2^_1, 0.05 _= 3.841

To test the allelic relationship of Huaai 11 with other dwarfing genes in barley, The F_1 _of Huaai 11 × Monker that is a popular commercial cultivar developed in USA was crossed with four dwarf varieties, Himalaya (*br*, 65.7 cm), Aiganqi (*uzu*,71.7 cm), India dwarf (*sdw1*, 61.3 cm) and Maris Mink (*denso*, 69.2 cm).

All abovementioned generations and their parents were grown on the Experimental Farm of Huazhong Agricultural University, Wuhan. A randomized complete block design with three replications was adopted. The materials were planted in five-row plots with a row length of 1.1 m.

To map the dwarfism gene in Huaai 11, a population consisting of 122 doubled-haploid (DH) lines was developed from a cross between a common feed barley cultivar Huadamai 6 and Huaai 11 using anther culture in this study. The DH population and parents were planted on the Experimental Farm of Huazhong Agricultural University, Wuhan, China. The field trials were conducted following a randomized complete block design with three replications in 2006, 2007 and 2008, respectively. Each of the DH and parental lines were grown in three rows in a plot of 0.6 × 1.5 m^2^.

Height of plants before ripening was measured in the field from soil surface to top of the main culm (with the spike). The height was calculated as the mean of the twelve plants (in three replicates, four plants from each replicate were measured).

### Extraction of genomic DNA

The leaves from each doubled-haploid (DH) lines and parents were collected and frozen for DNA extraction. The cetyltrimethylammonium bromide (CTAB) method was used to extract genomic DNA from about 0.6-1.0 g of young leaf-tissue of each accession [25]. The quality of DNA was checked using 0.8% agrose gel electrophoresis, and the DNA concentration was measured using spectrophotometer. Twelve extremely tall lines and twelve short lines were selected from the DH population to construct two DNA pools.

### SSR genotyping and mapping analysis

Three-hundred and six SSR markers distributed on all seven barley chromosomes were used to screen polymorphism between the two parental lines. The polymorphic SSR markers were further used to analyze the two DNA pools. The polymorphic markers between the two bulks were used to genotype the 122 individuals from the DH population. Genotyping data of 122 DH lines were given in Additional file 1(Table S1).

Polymerase chain reaction (PCR) was carried out in a final volume of 15 μL, containing 3 μL of the 20 ng/μL genomic DNA, 1.5 μL of 10× PCR buffer (with 15 mM Mg^2+^), 0.3 μL of 10 mM dNTP mixture, 2.0 μL of a 2.5 μM solution of the forward and reverse primers, and 0.6 units of *Taq *DNA polymerase (TakaRa Biotechnology, Dalian, China). DNA amplifications were performed in a thermocycler using the following touchdown PCR protocol: 1 cycle of 3 min at 94°C, followed by 15 cycles 94°C for 30 sec, 30 sec at 60°C (decreasing 1°C per cycle), 45 sec at 72°C. Another 25 cycles of 30 sec at 94°C, 30 sec at 50°C, 45 sec at 72°C. The reaction ended with a 5 min extension at 72°C. PCR product was separated on separated on 6% denaturing polyacrylamide gel and visualized using silver staining.

Linkage map was constructed using the software MAPMAKER [26] and the genetic distance (centimorgan, cM) was derived using Kosambi function [27].

## Results

### Inheritance of the dwarf gene in Huaai 11

**Figure 1:**
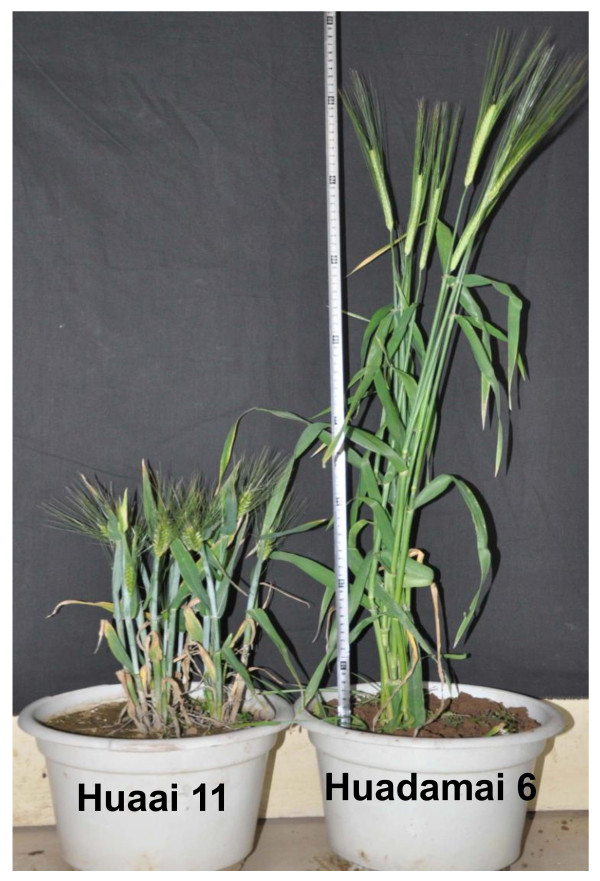
**Photo of the dwarf Huaai 11 and cultivar Huadamai 6 plants**.

Huaai 11 was discovered from the barley landrace Daofu Bai Qing Ke in 1993. Plant height of Huaai 11 is about 40 cm which is significant shorter than Huadamai 6 (85 cm) (Figure 1). Huaai 11 was crossed with nine tall varieties, Monker, Mpyt, Zhenongda 3, Zaoshu 3, Advance, Huadamai 1, Huadamai 6, Hyproly and Ris01508. All the F_1 _plants were tall. Distribution of plant height in all F_2 _populations are discontinuous, the distribution curves are dropped to valley at 60 cm, the plant equal or higher than 60 cm was classified as tall, while less than 60 cm as dwarf. Both the tall and dwarf plants appeared in all the F_2 _populations with a 3:1 segregation ratio for tall : dwarf (plant height ≥ 60 cm : plant height < 60 cm), suggesting that the dwarf trait in the Huaai 11 is controlled by single recessive gene. This gene is designated as *btwd1*.

**Figure 2:**
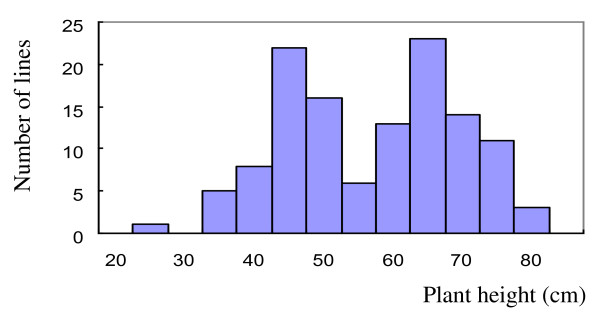
**Frequency distribution of plant height of the Huadamai 6/Huaai 11 DH population in 2007-2008**.

The DH population was constructed from the crosses between Huadamai 6 and Huaai 11 varieties. The averages of plant height of Huadamai 6 and Huaai 11 were 82.8 cm and 39.8 cm in three years, respectively. Figure 2 shows the distribution of plant height in the DH population in year 2007-2008. Plant height of the DH lines showed a bimodal distribution, ranging from 25 to 80 cm (Figure 2). The segregation ratio between tall and dwarf is 1:1.

### Allelic relationship between *btwd1 *and other dwarfing genes in barley

Plant height of Huaai 11 (40 cm) is much shorter than that of several other dwarf varieties such as Himalaya (*br*, 65.7 cm), Aiganqi (*uzu*, 71.7 cm), India dwarf (*sdw1*, 61.3 cm) and Maris Mink (*denso*, 69.2 cm). To test whether Huaai 11 shares the same allele with those four dwarf varieties, the F_1 _of Huaai 11 × Monker was crossed with those four varieties. All progeny in the four crosses are tall (Table 3), suggesting that the gene *btwd1*controlling plant height in the Huaai 11 is nonallelic with the genes in these four dwarf varieties.

**Table 3:** Segregation of plant height in the test crosses between F_1 _of Huaai 11 × Monker and four dwarf varieties

Cross	Generation	No. of total plants	No. of dwarf plants (< 60 cm)	No. of high plants (≥60 cm)	**χ**^2 ^**(1:1)**
(Huaai11/Monker)/Huaai 11	BC1	135	62	73	0.90
(Huaai11/Monker)/Aiganqi	TC1	119	0	119	0
(Huaai 11/Monker)/Himalaya	TC1	157	0	157	0
**(**Huaai 11/Monker)/Indian dwarf	TC1	117	0	117	0
(Huaai 11/Monker)/Maris Mink	TC1	143	0	143	0

χ^2^_1, 0.05 _= 3.841

### Molecular mapping of the dwarf gene *btwd1 *in Huaai 11

**Figure 3:**
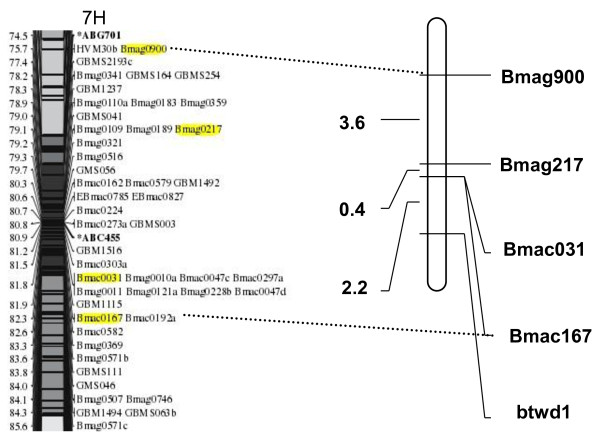
**Partial linkage map of chromosome 7H with the dwarfing gene *btwd1***. The chromosome 7H map was cited from Varshney et al. [35] for comparison

Out of the 306 SSR primers screened, ninety-six are polymorphic between parental lines Huadamai 6 and Huaai 11. The polymorphic SSR markers were used to test the two DNA pools for polymorphism. Four SSR markers, Bmac167, Bmac031, Bmag217 and Bmag900 located on chromosome 7H were polymorphic between the two DNA pools. Polymorphism was further confirmed using the individuals that were used to construct the two DNA pools, suggesting that the gene *btwd1 *controlling plant height of Huaai 11 was located on chromosome 7H. The four polymorphic markers were used to analyze the 122 individuals of DH population. Linkage analysis between SSR markers and plant height found that the dwarfing gene was located on the long arm of chromosome 7H, associated with the marker Bmac167 and Bmac031 at a genetic distance of 2.2 cM (Figure 3).

## Discussion

Utilization of dwarfing genes in barley breeding programs has greatly increased barley yields, particularly in Asia and Europe [28]. In barley, more than 30 types of dwarfs or semidwarfs have been reported. However, because pleiotropic effects of dwarf genes cause some other undesired agronomic traits such as reduced weight and yield [29,30], a few have been exploited in barley breeding. The plant architecture of newly discovered dwarf germplasm Huaai 11 consisted of desirable agronomic traits such as shortened stature and early maturity. It is a new source of dwarfs for broadening the genetic base of dwarfism. The segregation ratio (3:1) of tall : dwarf in the all crosses in two years suggested that dwarfism in Huaai 11 is controlled by a single recessive gene, *btwd1*.

The allelism test of Huaai 11 with four dwarf varieties, Himalaya (*br*), Aiganqi (*uzu*), India dwarf (*sdw1*) and Maris Mink (*denso*) showed that all progeny derived from test crosses between the F_1 _of Huaai 11 × Monker and those four varieties are tall (Table 3). Since the dwarfism of Huaai 11 is controlled by a Mendelian recessive allele, the F_1 _of Huaai 11 × Monker was tall as the tall parent. If the Huaai 11 is allelic with one of the four dwarf varieties tested here, the progeny will be expected to have tall : dwarf ratio of 1:1. No dwarf plants from the four crosses were observed, suggesting that the gene *btwd1 *controlling plant height in the Huaai 11 is nonallelic with the gene *br*, *uzu*, *sdw1 *and *denso*.

In barley, most of the dwarf and semi-dwarf genes were mapped on the chromosome 2H, 3H and 4H. Tsuchiya reported that two loci, the *br1 *locus on the short arm of chromosome 7 (7H) and *br2 *on the short arm of chromosome 4 (4H) [31,32]. Recently, Yu et al. mapped a semi-dwarfing QTL PH-7 in the ZAU7 on chromosome 7HL [28]. In our study, the dwarfing gene *btwd1 *was mapped on the long arm of chromosome 7H. The chromosome location of *btwd1 *is different from the *uzu *(3HL) [9,33,34], *sdw1/denso *(3HL) [18,19], which further demonstrated that the *btwd1 *in Huaai 11 is nonallelic with *br2*, *uzu sdw1/denso *genes. The *br1 *was mapped on 7HS, while the *btwd1 *was mapped on 7HL, indicating that they belong to different locus, and are non-allelic. The QTL PH-7 was located between the markers bPb4541 and bPb3107 on chromosome 7HL [28]. Examining the 7H linkage maps of Varshney et al. [35] and Yu et al. [28] revealed that QTL PH-7 is close to the distal of the 7HL, far away from centromere, while the *btwd1 *in Huaai 11 is linked with Bmac167 that is close to the centromere of 7H, suggesting that the *btwd1 *in Huaai 11 is different from QTL PH-7.

Most barley cultivars developed in China are dwarfs and semi-dwarfs since 1950 [2]. The study on origin of dwarfing genes used in barley breeding in China indicated that 68.4% of dwarf and semi-dwarf barley cultivars were derivatives of Chibadamai, Xiaoshanlixiahuang, Cangzhouluodamai, Aiganqi, Zhepi 1 and Yanfu Aizao 3 [2]. Allelism test indicated that Chibadamai, Xiaoshanlixiahuang, Cangzhouluodamai, and Aiganqi carried the same dwarfing gene *uzu *on 3HL [9,33,34]. Zhepi 1 and Yanfu Aizao 3 likely are mutations at the *sdw1 *locus [2]. The dwarfing gene *btwd1 *in Huaai 11 is non-allelic with the *uzu *and *sdw1 *which have widely been used in China, and provides barley breeders with a new gene in China. Quite often, when breeders use the dwarfing gene to reduce plant height, due to the confounded effects of other loci controlling plant height and environmental effects, not all plant carrying the dwarfing gene can be easily identified [28]. Thus, the linked SSR markers identified in the present study can provide a useful marker-assisted selection tool to transfer the dwarfing gene *btwd1 *into elite barley germplasm.

## Conclusions

Our study indicated that the dwarfing gene *btwd1 *in Huaai 11 is non-allelic with the *uzu *and *sdw1 *which have widely been used in China, and provides barley breeders with a new gene in China. Linkage analysis located the gene *btwd1 *gene (*btwd1*) onto the long arm of chromosome 7H, and it is closely linked to Bmac031 and Bmac167 with genetic distance of 2.2 cM.

## Authors' contributions

XFR is the major executive person in this study, including phenotyping of plant height, marker genotyping and statistical analysis. WWG assisted in phenotyping and marker genotyping. DFS and GLS conceived the design of this study, coordinated the experiments, and wrote the manuscript. CDL produced the Huadamai 6 and Huaai 11 DH population using the anther-culture method. All authors have read and approved the final manuscript.

## Supplementary Material

Additional file 1 **Table S1**. Genotyping data of 122 DH lines (*A: Plant height < 60 cm; B: Plant height ≥60 cm in btwd1). Click here for file
