# Supplementary material for: Inheritance and identification of molecular markers associated with a novel dwarfing gene in barley
Source: BMC Genet. 2010 Oct 8;11:89. doi: 10.1186/1471-2156-11-89 (PMC2959052; doi:10.1186/1471-2156-11-89)
Supplement: Additional file 1 — Table S1. Genotyping data of 122 DH lines (*A: Plant height < 60 cm; B: Plant height ≥60 cm in btwd1). [file 1471-2156-11-89-S1.DOC]

Table S: Genotyping data of 122 DH lines

| Line | Bmag900 | Bmag217 | Bmac167 | Bmac031 | Btwd1* | Line | Bmag900 | Bmag217 | Bmac167 | Bmac031 | Btwd1* | Line | Bmag900 | Bmag217 | Bmac167 | Bmac031 | Btwd1* |
| --- | --- | --- | --- | --- | --- | --- | --- | --- | --- | --- | --- | --- | --- | --- | --- | --- | --- |
| 1 | B | B | A | A | A | 42 | B | B | B | B | B | 83 | B | B | B | B | B |
| 2 | A | A | A | A | A | 43 | A | B | B | B | B | 84 | B | B | B | B | B |
| 3 | B | B | B | B | B | 44 | B | B | B | B | B | 85 | A | A | A | A | A |
| 4 | A | A | A | A | A | 45 | A | A | A | A | A | 86 | B | B | B | B | B |
| 5 | A | A | A | A | A | 46 | B | B | B | B | A | 87 | A | A | A | A | A |
| 6 | B | B | B | B | B | 47 | B | B | B | B | B | 88 | B | B | B | B | A |
| 7 | B | B | B | B | B | 48 | A | A | A | A | A | 89 | A | A | A | A | A |
| 8 | A | A | A | A | A | 49 | A | A | A | A | A | 90 | B | B | B | B | B |
| 9 | B | B | B | B | B | 50 | B | B | B | B | B | 91 | A | A | A | A | A |
| 10 | A | A | A | A | A | 51 | B | A | A | A | A | 92 | A | A | A | A | A |
| 11 | B | B | B | B | B | 52 | B | B | B | B | B | 93 | B | B | B | B | B |
| 12 | B | B | B | B | B | 53 | B | B | B | B | B | 94 | A | A | A | A | A |
| 13 | A | A | A | A | A | 54 | B | B | B | B | B | 95 | B | B | B | B | B |
| 14 | B | B | B | B | B | 55 | A | A | A | A | A | 96 | A | A | A | A | A |
| 15 | B | A | A | A | A | 56 | B | B | B | B | B | 97 | B | B | B | B | B |
| 16 | B | B | B | B | B | 57 | A | A | A | A | A | 98 | A | A | A | A | A |
| 17 | A | A | A | A | A | 58 | B | B | B | B | B | 99 | B | B | B | B | B |
| 18 | A | A | A | A | A | 59 | A | A | A | A | A | 100 | B | B | B | B | B |
| 19 | B | B | B | B | B | 60 | A | A | A | A | A | 101 | B | B | B | B | B |
| 20 | A | A | A | A | A | 61 | B | B | B | B | B | 102 | B | B | B | B | B |
| 21 | B | B | B | B | B | 62 | B | B | B | B | B | 103 | A | A | A | A | A |
| 22 | B | B | B | B | B | 63 | B | B | B | B | B | 104 | B | B | B | B | B |
| 23 | B | B | B | B | B | 64 | B | B | B | B | B | 105 | B | A | A | A | A |
| 24 | B | B | B | B | A | 65 | A | A | A | A | A | 106 | B | B | B | B | B |
| 25 | B | B | B | B | A | 66 | B | A | A | A | B | 107 | A | A | A | A | A |
| 26 | B | B | B | B | B | 67 | B | B | B | B | B | 108 | B | B | B | B | B |
| 27 | B | B | B | B | B | 68 | B | A | A | A | A | 109 | B | B | B | B | B |
| 28 | B | B | B | B | B | 69 | A | A | A | A | A | 110 | B | B | B | B | B |
| 29 | B | B | B | B | B | 70 | B | B | B | B | B | 111 | A | A | A | A | A |
| 30 | A | A | A | A | A | 71 | B | B | B | B | B | 112 | A | A | A | A | A |
| 31 | B | B | B | B | B | 72 | B | B | B | B | B | 113 | B | B | B | B | B |
| 32 | A | A | A | A | A | 73 | B | B | B | B | B | 114 | A | A | A | A | A |
| 33 | B | B | B | B | B | 74 | B | B | B | B | B | 115 | A | B | B | B | B |
| 34 | B | B | B | B | B | 75 | A | A | A | A | A | 116 | A | A | A | A | A |
| 35 | A | A | A | A | A | 76 | B | B | B | B | B | 117 | B | B | B | B | B |
| 36 | B | B | B | B | B | 77 | B | B | B | B | B | 118 | B | B | B | B | B |
| 37 | A | A | A | A | A | 78 | A | A | A | A | A | 119 | B | B | B | B | B |
| 38 | A | A | A | A | A | 79 | A | A | A | A | A | 120 | A | A | A | A | A |
| 39 | A | A | A | A | A | 80 | A | A | A | A | A | 121 | B | A | A | A | A |
| 40 | A | A | A | A | A | 81 | A | A | A | A | A | 122 | A | A | A | A | A |
| 41 | B | B | B | B | B | 82 | B | B | B | B | B |  |  |  |  |  |  |

*A: Plant height <60cm; B: Plant height ≥60cm in btwd1.
